# Inotropes for Preterm Infants: 50 Years on Are We Any Wiser?

**DOI:** 10.3389/fped.2018.00088

**Published:** 2018-04-06

**Authors:** Aisling A. Garvey, Elisabeth M. W. Kooi, Eugene M. Dempsey

**Affiliations:** ^1^Department of Paediatrics and Child Health, Neonatal Intensive Care Unit, University College Cork, Cork, Ireland; ^2^INFANT, Irish Centre for Fetal and Neonatal Translational Research, University College Cork, Cork, Ireland; ^3^Division of Neonatology, Beatrix Children’s Hospital, University Medical Center Groningen, University of Groningen, Groningen, Netherlands

**Keywords:** neonatology, hypotension, inotropes, preterm infants, blood pressure, end organ perfusion

## Abstract

For almost half a century, inotropes have been administered to preterm infants with the ultimate goal of increasing their blood pressure. A number of trials, the majority of which focused on dopamine administration, have demonstrated increased blood pressure following inotrope administration in preterm infants and have led to continued use of inotropes in our neonatal units. We have also seen an increase in the number of potential agents available to the clinician. However, we now know that hypotension is a much broader concept than blood pressure alone, and our aim should instead be focused on improving end organ perfusion, specifically cerebral perfusion. Only a limited number of studies have incorporated the organ-relevant hemodynamic changes and long-term outcomes when assessing inotropic effects in neonates, the majority of which are observational studies or have a small sample size. In addition, important considerations, including the developing/maturing adrenergic receptors, polymorphisms of these receptors, and other differences in the pharmacokinetics and pharmacodynamics of preterm infants, are only recently being recognized. Certainly, there remains huge variation in practice. The lack of well-conducted randomized controlled trials addressing these relevant outcomes, along with the difficulty executing such RCTs, leaves us with more questions than answers. This review provides an overview of the various inotropic agents currently being used in the care of preterm infants, with a particular focus on their organ/cerebral hemodynamic effects both during and after transition.

## Introduction

For almost half a century, inotropes have been administered to preterm infants with the ultimate goal of increasing mean arterial blood pressure (MBP). A number of trials, the majority of which focused on dopamine, have demonstrated increased MBP following inotrope administration in preterm infants. We have recently seen an increase in the number of potential inotropic agents available to the clinician with tailored therapeutic regimes advocated (1).

Continued reliance on MBP to initiate therapy and guide response remains the norm (2). However, hypotension is a much broader concept. A limited number of studies have incorporated organ-relevant hemodynamic changes and long-term outcomes when assessing inotropic effects, the majority of which are observational studies or have been limited by small sample size. In addition, important considerations including the maturing adrenergic receptors, polymorphisms, and other differences in the pharmacokinetics and pharmacodynamics of preterm infants are only recently being considered (3).

The lack of well-conducted RCTs addressing relevant outcomes, along with the difficulty in executing such trials (4), leaves us with more questions than answers. This review provides an overview of the various inotropic agents currently being used in the care of preterm infants, with a particular focus on hemodynamic effects, challenges in safe administration and why it is difficult to conduct RCTs in this area.

## Dopamine and Dobutamine

Few large RCTs have been performed investigating the effect of dopamine and/or dobutamine in preterm infants with hypotension or signs of low organ perfusion, but numerous observational studies and reviews have been published (5–7).

Dopamine is the most studied inotrope in preterm infants. It increases MBP (8–10) through its inotropic and vasopressor qualities. Dopamine is the direct precursor of noradrenaline (NA). It positively stimulates the α- and β-adrenoceptors, inducing both vascular smooth muscle and heart muscle contraction. The effects on cardiac output (CO) and end organ flow remain largely unanswered but are probably dose specific, with higher doses potentially inducing a significant increase in SVR which may attenuate its positive inotropic effect, highlighting the potential inotrope/afterload imbalance associated with some inotropes (8, 11). Dopamine administration not only results in increased serum dopamine levels but also in NA and epinephrine levels (12, 13). In these infants, endogenous catecholamine levels may already be high after birth (14).

Dobutamine is a synthetic catecholamine, stimulating β1-receptors, increasing CO, and reducing smooth muscle contraction resulting in peripheral vasodilation and a lower MBP increase when compared with dopamine (9, 10). It has been suggested as an alternative to dopamine; however, the different mechanisms of action might make it more suitable for certain disease states in the preterm infant.

Various parameters have been studied for the assessment of end organ blood flow. CO, mainly left ventricular output (LVO), has been described in a number of trials. A meta-analysis (15) comparing dopamine to dobutamine in hypotensive preterm infants concluded that dopamine led to a greater increase in MBP, whereas dobutamine had a greater effect on LVO. Clinically relevant outcomes such as IVH, PVL, or mortality were no different. An RCT including 41 preterm infants with low SVC flow (<41 ml/kg/min) were randomized to either dopamine or dobutamine after receiving a volume bolus. The results showed that although dobutamine resulted in a lower MBP, it had a significantly larger increase in SVC flow than dopamine (mean, +9.9 versus −3.2 ml/kg/min, *P* = 0.02). 40% failed to increase/maintain SVC flow in response to either agent and no differences in mortality or morbidity were found (16). More recently Bravo et al. randomized 28 preterm infants with low SVC to either dobutamine or placebo (17). The majority of infants achieved and maintained an SVC flow ≥ 41 ml/kg/min after intervention and infants treated with dobutamine (*n* = 16) showed a higher heart rate (HR) and improved base excess compared with those treated with placebo (*n* = 12).

Two studies have assessed the effect on surrogate markers of cerebral blood flow. Using xenon techniques, Lundstrøm et al. found dopamine administered at 5 μg/kg/min resulted in increased BP and LVO, but no change in cerebral blood flow, in preterm infants with an MBP between 29 and 40 mmHg (18). This would suggest that dopamine results in a degree of vasoconstriction, either directly from its effect on cerebral vasculature or from a compensatory autoregulatory effect from the increase in systemic BP. This lack of increased cerebral perfusion was also suggested from fetal sheep studies where the dopamine-induced increased BP resulted in a cerebral autoregulatory α-adrenergic vasoconstrictive response, maintaining cerebral blood flow (19). However, in a subsequent RCT comparing dopamine versus epinephrine in preterm infants with low BP, Pellicer and colleagues measured the effects on cerebral oxygenation as a surrogate for cerebral perfusion (20). They found no difference in the increase in cerebral oxygenation, suggesting an increase in cerebral perfusion from both drugs.

While there are few RCTs addressing end organ perfusion there are numerous observational studies reporting effects including right ventricular output (RVO), LVO, pulmonary pressures, and cerebral blood flow. One report on the effect of dopamine on right ventricular performance included hypotensive preterm neonates in whom right ventricular end systolic volume decreased approximately 30%, whereas right ventricular end diastolic volume did not change. Ejection fraction therefore increased and RVO increased from 90 to 112 ml/kg/min (21). Specifically in preterm infants with septic shock, in whom either dopamine or dobutamine was commenced, RVO and HR increased, but LVO and organ flow did not. After analyzing subgroups, both drugs did not seem to significantly increase both LVO and RVO. The increase in HR was mainly seen after dopamine infusion (22). The effect on pulmonary artery pressure was described by Liet et al. in 19 preterm ventilated infants (4 ± 3 days of life), with a patent ductus arteriosus (PDA), showing an overall increase in arterial pulmonary pressure following dopamine administration (23).

Seri et al. found an increased BP and urinary output in preterm infants (<2 days) following dopamine administration, but no change in mesenteric and cerebral perfusion indices (24). Contradictive results have been reported on the effect of dopamine on cerebrovascular autoregulation in infants. Wong et al. found an increased potential for cerebral flow and metabolism coupling, suggesting an improved blood supply in response to demand (25). Contrary to these findings, Eriksen et al. found dopamine had an adverse effect on cerebrovascular autoregulation in preterm infants (26). A meta-analysis of predominantly observational studies investigating the effect of dopamine showed an overall increased cerebral blood flow (27). They concluded from 8 small studies (*n* = 5–36) containing a total of 153 preterm infants, that the increase in CBF was greater in hypotensive than in normotensive preterm infants. This effect was not repeated in newborn piglets, in which dopamine neither increased CO nor cerebral blood flow (28). Using NIRS as a method to assess cerebral perfusion in 71 preterm infants without a PDA, a modest increase in cerebral oxygen saturation was seen, both after volume and after dopamine administration (29). Volume administration did not alter cerebral oxygen extraction in a small population of preterm infants with clinical signs of poor perfusion (30). The previously mentioned meta-analysis showed no statistically significant differences in adverse neurological outcome between dopamine and dobutamine (three studies; *N* = 118; *r* = −0.13; 95% CI = −0.31 to 0.059) (27).

## Adrenaline and NA

Adrenaline is typically used as a second or third line agent in the management of hypotension (2). One RCT compared adrenaline to dopamine in preterm infants <32 weeks gestation with low BP. MBP showed a significant increase from baseline throughout the first 96 h with no differences between groups but adrenaline caused a greater increase in HR and glucose levels more likely to need insulin. They also had higher plasma lactate and lower bicarbonate and base excess values (31). This increase in serum lactate may limit the use of sequential serum lactate measurements to monitor the changes in the perfusion status. As mentioned previously, both low/moderate-dose dopamine and low-dose adrenaline increased cerebral perfusion, as indicated by the increase in both CBV and HbD measured by NIRS (20).

Noradrenaline is a naturally occurring sympathomimetic amine which exerts its cardiogenic effects through activation of α- and β-adrenoceptors. NA poorly stimulates the vascular β-adrenoceptors and has a greater effect on peripheral vascular resistance, making it potentially useful in profound hypotension and septic shock (32). Rios et al. examined trends in treatment of neonatal hypotension and found that NA was used in 0.6% of admissions (33). To date, there are no RCTs assessing the efficacy of NA in neonatal cardiovascular compromise, and the available evidence is predominantly from observational trials. Concerns around NA’s potentially potent vasoconstrictor effect has resulted in limited use in preterm infants as it could lead to decreased CO and as a consequence poor tissue perfusion (34, 35). Liem retrospectively examined the use of NA in babies born 29–39 weeks gestation. NA succeeded in increasing BP in all babies with hypotension but half required another agent to achieve normotension (36). Rowcliff et al. retrospectively examined the use of NA in infants born less than 32 weeks gestation and found it was effective in increasing MBP. Although tachycardia could be a side effect, no other adverse effects were identified (37). Barrington et al. report the effects of NA in 30 preterm infants with septic shock, highlighting an increase in MBP and urine output following administration (38).

In adults and older children with septic shock, NA is the inotrope of choice due to the presence of low SVR (39). NA administration was also associated with increased urine output and decreased lactate suggesting an improvement in cardiac function and tissue perfusion (40). Derleth used NA in 29 infants born between 22 and 38 weeks gestation with septic shock and found less incidence of PVL in those treated with NA suggesting that despite its vasoconstrictor properties, NA succeeds in maintaining a steady cerebral and myocardial blood flow (41).

Animal studies have shown that NA increases SVR and pulmonary blood flow and therefore improves outcome in pulmonary hypertension (42). Tourneux et al. examined this effect in infants born >35 weeks gestation with PPHN and demonstrated improved lung and cardiac function (43). A recent adult study found NA was associated with more enterocyte damage, which might be an important factor to consider in preterm infants (44).

## Vasopressin

Arginine vasopressin (AVP) is a naturally occurring neuropeptide secreted by the posterior pituitary gland. AVP has little or no inotropic or chronotropic effect, instead it exerts its actions on the vasculature V1 receptors resulting in arterial vasoconstriction and the V2 receptors of the renal tubule leading to reabsorption of renal free water. The majority of our evidence in preterm infants is from observational studies in refractory hypotension where administration of vasopressin results in an improvement in MBP, urinary output and HR without increasing lactate (45–51). Rios and Kaiser compared vasopressin to dopamine for treatment of hypotension in extremely low birth weight infants (52). Both were shown to be effective in increasing systemic BP, but vasopressin was associated with a reduction in PaCO_2_, surfactant use, and tachycardia.

Adult studies have demonstrated a reduced level of plasma vasopressin in patients with septic shock (53). This depletion of vasopressin is also evident in pediatric patients, in which small doses of vasopressin resulted in improved systemic BP and urine output (54, 55). Meyer et al. compared response to vasopressin in three infants with septic shock to three infants with non-septic shock. While vasopressin resulted in increased BP and urine output in both groups, the results were transient in the non-septic shock infants. Septic shock infants had a reduced HR and lactate level whereas the opposite occurred in the non-septic shock group (56). Many studies note a decrease in requirement of other inotropic agents following initiation of vasopressin to maintain/increase BP (47, 55). Vasopressin has also been shown to increase the sensitivity of vasculature to NA (57).

A potential side effect is poor end organ perfusion (58, 59). However, the majority of studies involving vasopressin document its use in refractory hypotension or septic shock in which end organ involvement is often present before initiation (60). There are no data regarding its effect on CO and cerebral blood flow in preterm infants.

Vasopressin has potential benefit in the treatment of PPHN. In animal studies, vasopressin results in the reduction of pulmonary arterial pressures and the pulmonary:systemic ratio (61). One small observational study in 10 infants with PPHN found that administration of vasopressin resulted in improved oxygenation, BP, urine output, and a reduced iNO requirement (62). Similar findings were noted in patients with congenital diaphragmatic hernias where vasopressin also resulted in improved left ventricular function and oxygenation index (63). The role of vasopressin in the management of circulatory instability in preterm infants remains to be determined.

## Milrinone

Milrinone is a selective phosphodiesterase III inhibitor in cardiac myocytes and vascular smooth muscle. It has potent vasodilator and inotropic effects leading to a reduction in afterload by decreasing pulmonary vascular resistance (64–66). Milrinone is excreted by the kidneys with little or no metabolism (67), therefore, plasma concentration largely depends on renal function. Paradisis et al. demonstrated a longer half-life in preterms (68) and Giaccone et al. found newborns with PPHN had reduced clearance (69).

Animal studies have demonstrated an improvement in right ventricular function and pulmonary vascular resistance (70, 71). These findings are consistent with studies involving children post-cardiac surgery (64, 72). However, the cAMP levels are decreased in the newborn myocardium, thus newborns may be less sensitive to the effect of milrinone (73, 74).

Paradisis conducted one of the few RCTs comparing an agent to placebo in preterm infants (75). Ninety infants born <30 weeks gestation were randomized to either milrinone or placebo. They showed no significant difference in SVC flow between the two groups. Jain et al. utilized targeted neonatal echocardiography to measure LVO in preterm infants post PDA ligation. Babies with LVO < 200 ml/kg/min received IV milrinone, and this group was compared with a historic cohort of babies with the same echocardiographic findings post ligation. Those that received milrinone had significantly lower ventilation failure rates, oxygenation failure, and need for inotropic support or steroids, suggesting improved cardiovascular stability (76). Previous studies have advised caution with administration of milrinone as it transiently reduced MBP (77, 78) although little is mentioned on end organ perfusion. Paradisis found significantly lower MBP and a higher HR at 7 and 10 h compared with placebo but no significant difference in hypotension (defined as an MBP < 24 mmHg). It is important to highlight that this study enrolled all babies at risk of low SVC flow as opposed to babies with established hypotension or low-flow states.

Due to its effect on PVR, it may have a useful role in the treatment of PPHN. Samiee-Zafarghandy et al. looked at milrinone use in Neonatal Intensive Care Units (NICU) in North America (79). Milrinone was used in 0.4% of infants, and the main indication for use (42%) was PPHN. There are a number of observational studies regarding its use in PPHN (77, 78, 80, 81). Additional use of milrinone resulted in a reduction in oxygenation index and mean airway pressure. In babies with iNO resistant PPHN, milrinone resulted in improved LVO, RVO, and ultimately a reduction in iNO requirement (82). RCTs in older children with septic shock demonstrated an improved CO post-milrinone administration (83); however, such studies are lacking in preterm infants.

## Practical Administration Challenges

In addition to the pharmacodynamics, pharmacokinetics, and pharmacogenetics of inotrope use in preterm infants, delivery kinetics play an important role and must be considered when observing effects. This is especially the case in low-flow rate continuous IV drugs, such as vasoactive drugs for preterm infants, where flow rates of 0.1–1 ml/h are not uncommon (84). These very low-flow rates combined with relatively significant dead space volumes lead to longer time to onset and steady state of intended doses, and therefore unintended highly variable and unpredictable doses may occur after dosing changes (85). The longer duration for the actual intended dose to be delivered, for drugs with narrow therapeutic indices, can result in significant, unrecognized, and potentially hazardous situations (84).

Several non-patient-specific factors have been studied that might influence this flow rate variability during low-flow rates, which are summarized to the set flow rate, hydrostatic pressure changes and compliance of the complete IV administration system “from pump to patient,” and type of substances administered (86).

## What is Next?

Despite 50 years of use, we still have little evidence regarding end organ perfusion and outcome of the various drugs used for both hypotension and low-flow states in preterm infants. It remains unclear whether cerebral perfusion is increased, unaltered, or reduced with any inotrope use. The effects are most likely mediated by alterations in systemic BP, pulmonary vascular resistance, a direct effect on cerebral vasculature, altered cerebrovascular autoregulation, or a combination of all of the above. Utilization of more objective assessment methods may allow us to gain better insight into these effects (87).

Conducting trials of inotropes in preterm infants has proven challenging on a number of fronts. The majority of the RCTs to date have been conducted in the 1990s with few of these reporting clinically important end points (Table 1). Over the last 10 years, improvements in perinatal care such as antenatal steroid exposure and changes in ventilation management have seen a reduction in the incidence of low BP in preterm infants. Achieving timely informed consent has proved problematic, and as such enrollment has been difficult resulting in very few recent trials to inform the neonatal community. Two small pilot trials have been conducted comparing dopamine to vasopressin (20 infants) and dobutamine to placebo (28 infants). A recent randomized trial highlighted practical challenges, where 10 newborns from a potential population of 127 babies were enrolled across 16 sites (88, 89). A recent trial of cardiovascular compromises again highlighted challenges in enrollment in trials of cardiovascular support (90). It is clear that it is proving difficult to perform studies that require enrollment within a short-time window when newborn infants are unstable and often require prompt intervention (91). Until such issues are addressed, we will continue to utilize agents that may be of no benefit, or potentially have adverse consequences. Institutional review boards, regulatory authorities, funding agencies, and parental organizations need to be aware of these challenges, and an international approach to the problem is required. The recently established International Neonatal Consortium may have a very important role to play in highlighting these challenges and facilitating the conduct of high quality trials of investigational medicinal products, in particular inotropes, in the next number of years.

**Table 1:** Randomized control trials and measurement of end organ perfusion.

RCT	Agents	No. enrolled	Gestation (weeks)/birth weight (g)	LVO	RVO	SVC flow	Cerebral perfusion/blood flow	GI Perfusion	Urine output	Lactate
Roze et al. (8)	Dop versus Dob	20	<32	x						
Greenough et al. (9)	Dop versus Dob	40	<34							
Klarr et al. (10)	Dop versus Dob	63	≤34						x	
Osborn et al. (16)	Dop versus Dob	42	<30		x	x				
Chatterjee et al. (92)	Dop versus Dob	20	<32	x	x					
Hentschel et al. (93)	Dop versus Dob	20	25–36					x		
Ruelas-Orozco et al. (94)	Dop versus Dob	66	1,000–1,500							
Gill et al. (95)	Dop versus volume	39	<1,501							
Lundstrøm et al. (18)	Dop versus volume	36	<33	x			x			
Bravo et al. (17)	Dob versus placebo	127	<31			x	x			x
Cuevas et al. (96)	Dop versus placebo	49	700–2,000						x	x
Pellicer et al. (20)	Dop versus Adr	60	<32				x		x	x
Valverde et al. (31)										
Phillipos et al. (97)	Dop versus Adr	20	>1,750	x	x					
Rios and Kaiser (52)	Dop versus vasopressin	20	≤30						x	x
Paradisis et al. (75)	Milrinone versus placebo	90	<30		x	x	x			

*x, outcome has been reported; LVO, left ventricular output; RVO, right ventricular output; SVC, superior vena cava; GI, gastrointestinal; Dop, dopamine; Dob, dobutamine; Adr, adrenaline*.

## Author Contributions

All the authors have contributed by writing parts of the first draft, reviewing and adjusting the manuscript to its current form. All the authors agree with the final version of the article.

## Conflict of Interest Statement

The authors declare that the research was conducted in the absence of any commercial or financial relationships that could be construed as a potential conflict of interest.
