# Supplementary material for: Inotropes for Preterm Infants: 50 Years on Are We Any Wiser?
Source: Front Pediatr. 2018 Apr 6;6:88. doi: 10.3389/fped.2018.00088 (PMC5898425; doi:10.3389/fped.2018.00088)
Supplement: Supplementary file 1 [file table_1.docx]

Supplementary Material

**Inotropes for preterm Infants: Fifty years on are we any wiser?**

Aisling A Garvey, Kooi EMW, Dempsey EM.

*** Correspondence:** Corresponding Author: g.dempsey@ucc.ie

# Supplementary Data

| RCT | Agents | No. enrolled | Gestation (weeks)/ birth weight (grams) | LVO | RVO | SVC Flow | Cerebral perfusion/ blood flow | GI Perfusion | Urine output | Lactate |
| --- | --- | --- | --- | --- | --- | --- | --- | --- | --- | --- |
| Roze et al(8) | Dop v Dob | 20 | <32 | x |  |  |  |  |  |  |
| Greenough et al(9) | Dop v Dob | 40 | <34 |  |  |  |  |  |  |  |
| Klarr et al(10) | Dop v Dob | 63 | ≤34 |  |  |  |  |  | x |  |
| Osborn et al(15) | Dop v Dob | 42 | <30 |  | x | x |  |  |  |  |
| Chatterjee et al(86) | Dop v Dob | 20 | <32 | x | x |  |  |  |  |  |
| Hentschel et al(87) | Dop v Dob | 20 | 25-36 |  |  |  |  | x |  |  |
| Ruelas-Orozco et al(88) | Dop v Dob | 66 | 1000-1500g |  |  |  |  |  |  |  |
| Gill et al(89) | Dop v Volume | 39 | <1501g |  |  |  |  |  |  |  |
| Lundstrom et al(17) | Dop v Volume | 36 | <33 | x |  |  | x |  |  |  |
| Bravo et al(16) | Dob v Placebo | 127 | <31 |  |  | x | x |  |  | x |
| Cuevas et al(90) | Dop v Placebo | 49 | 700-2000g |  |  |  |  |  | x | x |
| Pellicer et al (19)  Valverde et al(30) | Dop v Adr | 60 | <32 |  |  |  | x |  | x | x |
| Phillipos et al(91) | Dop v Adr | 20 | >1750g | x | x |  |  |  |  |  |
| Rios et al(51) | Dop v Vasopressin | 20 | ≤30 |  |  |  |  |  | x | x |
| Paradisis et al(72) | Milrinone v Placebo | 90 | <30 |  | x | x | x |  |  |  |

**Supplementary Figure 1.** Randomised Control Trials and measurement of end organ perfusion. X, outcome has been reported; LVO, left ventricular output; RVO, right ventricular output; SVC, superior vena cava; GI, gastrointestinal; Dop, dopamine; Dob, dobutamine; Adr, Adrenaline..
